# Analysis of KIT gene mutations in patients with melanoma of the head and neck mucosa: a retrospective clinical report

**DOI:** 10.18632/oncotarget.25094

**Published:** 2018-05-01

**Authors:** Ullyanov Bezerra Toscano de Mendonça, Claudio Roberto Cernea, Leandro Luongo Matos, Roberto Rego Monteiro de Araujo Lima

**Affiliations:** ^1^ Department of Head and Neck Surgery, Instituto Nacional do Cancer do Rio de Janeiro, Rio de Janeiro, Brazil; ^2^ Department of Head and Neck Surgery, School of Medicine, University of São Paulo, Sao Paulo-SP, Brazil

**Keywords:** mucosal melanoma, KIT, c-kit, mutation, proto-oncogene

## Abstract

Unlike their cutaneous counterparts, head and neck mucosal malignant melanomas (HNMM) are more aggressive, and their prognostic markers have not been fully elucidated. This study, comprising 28 patients with HNMM, aimed to establish the relationship between different mutations and outcome, define the incidence of KIT mutations in HNMM, and identify the correlation among therapeutic options, histopathological findings, demographic data, and clinical response. Clinical analysis included patient characteristics, staging, primary and palliative treatments, and disease-free survival and overall survival (OS). Progression-free survival and OS were analyzed. Paraffin blocks were selected following histologic analyses, enabling DNA extraction. PCR amplification of exons 9, 11, 13, and 17, with different DNA concentrations, was performed. Patients were predominantly females (57%) and aged 27–85 years. All patients underwent surgery; 17 received adjuvant radiotherapy, and recurrences occurred in 82% patients. Oncologic mutations in KIT were found in 7 of 7 tumors, 3 in exon 9, 3 in exon 11, and 1 in exon 13. Predictive factors for recurrence were mitotic rate, vascular invasion, and perineural spread. There were no significant differences in DFS and OS according to KIT mutation. Our study results suggest that some patients might benefit from appropriate targeted therapy with kinase inhibitors.

## INTRODUCTION

The development of melanoma is a classic example of a neoplasm that progresses through different known stages. However, the key molecular event that triggers the progression of this neoplasm has still not been clarified, which explains why there is no specific therapy and why there have been so few advances in the multimodal therapy of this disease [1].

Somatic mutations in the KIT gene, which were identified in a small number of skin melanomas, appear to have a higher incidence in mucosal melanomas. Curtin *et al.* found mutations or an increase in the number of copies of the KIT in 39% of mucosal melanomas. Moreover, there was an increase in KIT protein expression in mucosal melanomas, which supports their role in the progression of this melanoma subtype [2]. Given the evidence of a possible pathogenic role of the KIT gene in a number of mucosal melanomas, including those of the head and neck, screening for KIT aberrations may have diagnostic value, and the gene may represent a therapeutic target in these patients [3].

The identification of activating mutations in the KIT gene in patients with mucosal melanoma is important to improve knowledge of tumor biology and the design of clinical research protocols with imatinib. Because mucosal melanoma of the head and neck is a rare condition, the frequency of KIT mutations has not been characterized in these tumors. The objective of the present study was to evaluate the frequency of KIT mutations and their prognostic value in a significant number of head and neck mucosal melanomas [4].

## RESULTS

Twenty-eight cases were included in the study. Summary information on each case is given in Table 1. Patient age ranged from 27 to 88 years, with a median of 59.5 years; 16 patients (57.1%) were women; 24 patients (85.7%) were white, and 4 patients were black. The majority of the tumors were classified as T4 (75%) and the majority of the cases were N0 or stage IV patients. Seven patients (25%) had undifferentiated primary tumors, 17 patients (60.5%) had tumors with a mitotic index higher than 10 mitoses/mm2, and only 8 patients (28.6%) exhibited amelanotic tumors.

**Table 1:** Summary of cases included in the study

Case	Gender	Age	Ethnicity	Treatment	Relapse	SG (months)	KIT
1	Female	69	White	CX + RXT	Yes	117	Wild
2	Female	66	White	CX + RXT	Yes	54	Wild
3	Female	38	White	CX	Yes	11	Wild
4	Female	88	White	CX + RXT	Yes	Still Alive	Wild
5	Female	64	White	CX + RXT	No	Still Alive	Mutated
6	Female	61	White	CX + RXT	No	Still Alive	Wild
7	Female	62	White	CX + RXT	Yes	27	Wild
8	Female	62	White	CX	Yes	29	Wild
9	Female	39	White	CX + RXT	Yes	Still Alive	Wild
10	Female	62	White	CX	No	Still Alive	Wild
11	Female	27	White	CX	Yes	10	Wild
12	Female	59	White	CX	Yes	Still Alive	Wild
13	Female	60	White	CX	Yes	8	Wild
14	Female	76	White	CX	Yes	11	Wild
15	Female	63	White	CX	Yes	14	Wild
16	Female	69	White	CX	Yes	14	Wild
17	Male	32	White	CX + RXT	Yes	19	Wild
18	Male	85	White	CX + RXT	Yes	43	Wild
19	Male	47	White	CX	No	Still Alive	Wild
20	Male	39	White	CX + RXT	Yes	64	Wild
21	Male	53	White	CX + RXT	Yes	99	Mutated
22	Male	52	White	CX + RXT	Yes	Still Alive	Mutated
23	Male	62	White	CX + RXT	Yes	27	Wild
24	Male	51	White	CX + RXT	No	Still Alive	Wild
25	Male	40	White	CX + RXT	Yes	15	Mutated
26	Male	81	Black	CX	Yes	60	Mutated
27	Male	69	Black	CX + RXT	Yes	21	Mutated
28	Male	76	Black	CX + RXT	Yes	11	Mutated

CX + RXT = Surgical resection followed by adjuvante radiotherapy, CX = Exclusive surgical resection, OS = Overall survival.

### Analysis of KIT gene mutations

**Figure 1:**
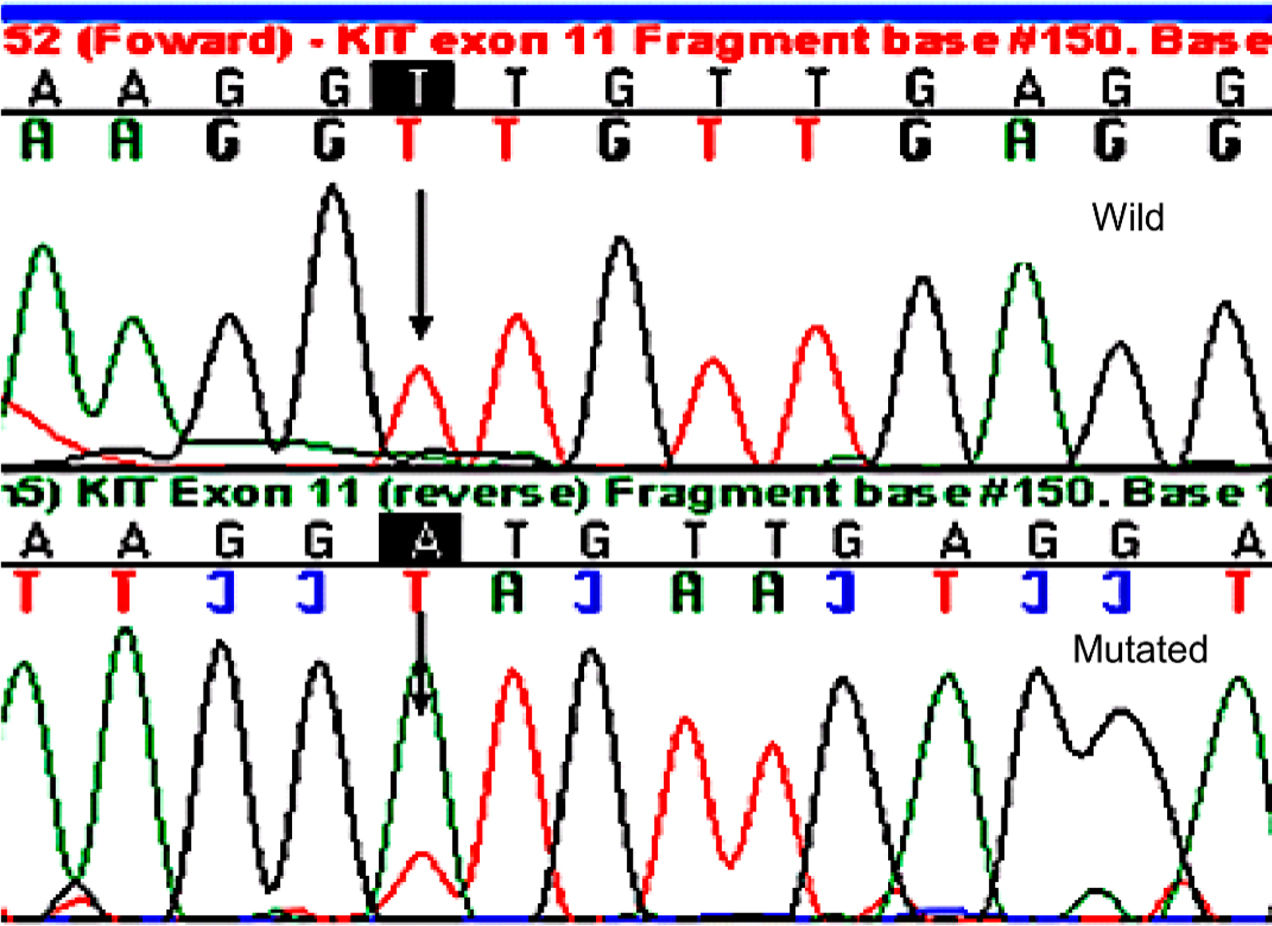
Chromatogram showing mutation in exon 11 of the gene KIT/L576P of a representative case (case 26)

**Figure 2:**
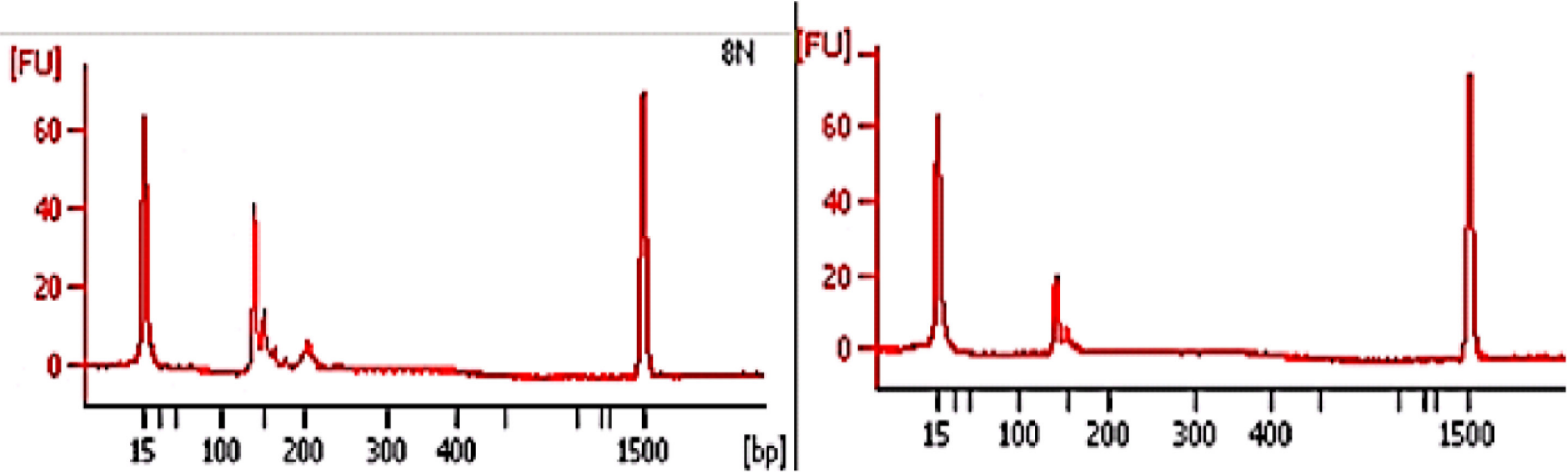
Electropherogram showing loss of heterozygosity of the KIT gene (HK 8810 Marker) (analysis using an Agilent 2100 bioanalyzer)

Analysis of KIT gene mutations was possible in all of the 28 cases studied; 7 patients had KIT mutations and 21 patients had wild-type KIT. KIT mutations were most frequently detected in exon 11 (42.8%) and exon 9 (42.9%). Table 2 describes all the mutations found. Figure 1 shows a chromatogram of a mutation in exon 11 of the KIT gene, and loss of heterozygosity is represented in Figure 2. There was no significant relationship between the clinical and demographic variables and the presence of the KIT mutation (*p* > 0.05; Table 3).

**Table 2:** Distribution of the KIT mutations found

Patient	Mutational status	Type of mutation	Note
5	Exon 11	V551I	Heterozygous
21	Exon 11	V551I	Heterozygous
22	Exon 13	L657F	Homozygous
25	Exon 9	L455M	Heterozygous
26	Exon 11	L576P	Heterozygous
27	Exon 9	S480F	Heterozygous
28	Exon 9	G499S	Heterozygous

**Table 3:** Clinical variables and their correlation with the KIT mutation

Variables	KIT		p^*^
	Wild *N* = 21 (75%)	Mutation *N* = 7 (25%)	
**Age**			
>60	12 (42.9%)	4 (14.3%)	0.666
<60	9 (32.1%)	3 (10.7%)	
**Sex**			
Female	12 (42.9%)	9 (32.1)	0.672
Male	4 (14.3%)	3 (10.7%)	
**Race**			
Whites	18 (64.3%)	6 (21.4%)	0.747
Blacks	3 (10.7%)	1 (3.6%)	
**Smoking**			
Yes	9 (32.1%)	4 (14.3%)	0.412
No	12 (42.9%)	3 (10.7%)	
**Alcohol use**			
Yes	5 (17.9%)	2 (7.1%)	0.581
No	16 (57.1%)	5 (17.9%)	
**Location**			
Nasosinusal	4 (14.3%)	4 (14.3%)	0.220
Oral cavity	17 (60.7%)	3 (10.7%)	

^*^Fischer’s exact test.

The DFS (disease-free-survival) rate for the cancer was 53.6% in 24 months and 37.5% in 60 months. The absence of adjuvant treatment and a mitotic index higher than 10 mitoses/mm2 were associated with a higher probability of death (*p* = 0.05, log-rank test). The presence of vascular invasion and angiolymphatic dissemination were also statistically significant (*p* = 0.04 and *p* = 0.02, respectively, log-rank test). In addition, there was a statistically significant relationship between recurrence and a mitotic index higher than 10 mitoses/mm2, vascular invasion, angiolymphatic dissemination and perineural dissemination (*p* = 0.05, *p* = 0.043, *p* = 0.008 and *p* = 0.034, respectively, log-rank test). There were no statistical differences between the groups, and the presence of mutation did not play a role, either in protecting or promoting relapse or death.

## DISCUSSION

Because of the rarity of this disease, only 28 cases were enrolled in the study. Kanda (2003) presented a study with a total of 54 cases from 3 different institutions, which demonstrates the rarity of the condition in the national context [5]. Therefore, much of the literature that exists on the subject addresses isolated cases and consists of retrospective analyses of series with relatively small sample sizes [6, 7].

Some peculiarities of the biological behavior of these tumors are extremely important: the anatomical location of the lesion, including its staging. Thus, some early-stage tumors may exhibit an aggressive behavior due to their location.

The historical results of HNMM treatment are disappointing. This soon led researchers to test new treatment strategies, such as the addition of chemotherapy or targeted therapy.

Defining prognostic parameters for HNMM is a much more complex task because the depth of invasion—the most important prognostic factor in skin melanomas—cannot be used due to the lack of histological points of reference similar to the papillary and reticular dermis.

Recent studies have evaluated the oncogenic role of KIT mutations in HNMM, as well as the benefits of therapy with tyrosine kinase inhibitors in these tumors. The results appear to be encouraging, showing significant benefits in survival time over chemotherapy and targeted therapy [8].

HNMM most frequently affects patients between the fifth and seventh decades of life, with more than 60% of patients belonging to this age group [9]. In this study, 16 (57%) patients were aged over 60. When we compared these 2 age groups, i.e., patients aged over 60 and those aged under 60, with regard to disease recurrence and mortality, there was no significant difference (*p* = 0.38 and *p* = 0.648).

According to some authors there are no differences in the incidence of the location of HNMM, i.e., the incidence of tumors of the sinuses of the face and nose is equivalent to that of tumors of the oral cavity [9, 10]. However, we found a higher incidence of tumors of the sinuses of the face (75%) in our sample. This is due to factors related to the treatment; considering that in INCa more complex tumors are treated, we believe this is due to the sample selected.

Retrospective series did not demonstrate a relationship between the primary site of the tumor and both survival and local control [11, 12]. This observation was confirmed in the present study. However, there are some reports of a poorer prognosis related to tumors originating in the sinuses of the face, probably because of the presence of locally advanced disease at diagnosis [13].

We used the TNM classification in the present study. The majority of tumors were classified as T4 (75%) and the majority of patients had N0 neck and were considered stage IV. We did not find any differences in OS and DFS (*p* = 0.899 and *p* = 0.523, respectively). In the present study, we observed a mitotic index higher than 10 mitoses per field in 60.7% of the patients. This is an important result as it suggests a greater aggressiveness in these tumors. It is interesting to highlight that after analysis of DFS and OS, the mitotic index was found to be an independent prognostic factor for both end-points. OS in patients with a mitotic index higher than 10 mitoses per field was 36.4% in 48 months versus 51.5% in the other group (*p* = 0.05). Thompson *et al.* (2003) studied 115 patients with HNMM and obtained similar results, i.e., a poorer prognosis in the group with a high mitotic index (*p* = 0.026) [14]. In a recent work published by Moreno *et al.* (2010), 95% of patients with a high mitotic index died from the HNMM, whereas patients with a mitotic index of less than 10 had a better response to systemic therapy [15]. In our sample, we found 8 cases (28.6%) of amelanotic tumors, all confirmed by immunohistochemistry. Data from the literature suggest greater aggressiveness in amelanotic tumors, this being the only histopathological factor related to survival; no patient was alive after 48 months, compared with 47.6% survival in patients with pigmented tumors [15]. Our results did not confirm these data, but we had an OS of 55% in 48 months in patients with pigmented tumors, compared with 18.8% in those with amelanotic tumors. The explanation accepted for a poorer prognosis in amelanotic tumors is that they are associated with a delayed diagnosis.

In the sample, it was not possible to compare surgical treatment with other therapy modalities, because all the patients were submitted to resection. We obtained free margins in 82.15% of the patients; however, the finding of free margins had no impact on survival. Patients with positive surgical margins were 21 times more likely to die due to therapeutic failure, but we did not find this relation in the sample [16].

In local recurrent disease, and in the absence of metastatic dissemination, surgical removal should be considered, but the extent of the resection should be carefully planned. The chance of a successful surgical removal is less than 25%, and the chances of distant metastases are increased [17].

The main motivation for performing this study was to investigate a marker with therapeutic potential for a disease that, although rare, is highly lethal. Moreover, there are few studies involving the research of KIT in HNMM, possibly as a result of the rarity of the disease.

The presence of KIT mutations in malignant melanomas has already been demonstrated in the literature, in particular in melanomas of the mucosa (21% of KIT mutations), acral melanomas (11% of KIT mutations), and skin melanomas caused by chronic exposure to sunlight (17% of KIT mutations) [2]. This suggests that KIT mutations have a role in the physiopathogeny and, therefore, are a potential therapeutic target in these subtypes of melanoma. In contrast, KIT mutations are rarely found in the largest subgroup of malignant melanomas, SM not associated with chronic exposure to sunlight: 1 in 100 cases studied by Willmore-Payne *et al.* (2005) [18]. Furthermore, phase II studies with imatinib in patients with SM, without KIT mutation analysis, were disappointing [19–21].

KIT mutations were evaluated in a selected group of patients with HNMM. Molecular analysis of KIT mutations was possible in 28 patients and showed mutations in 7 of them (25%). This is consistent with the discoveries of Antonescu *et al.* (2007), and Rivera *et al.* (2008), who detected mutations in the KIT gene in 3 out of 20 patients (15%) and in 4 out of 18 patients (22%) with mucosal melanomas of the anal region and of the oral cavity, respectively [22–24]. Thus, KIT mutations occur in up to 20% of mucosal melanomas, regardless of the location of the primary tumor. Three mutations were detected in exon 11, 3 mutations in exon 9, and 1 mutation in exon 13; this distribution with a higher frequency of mutations in exons 11 and 13 is in line with the literature [25, 26]. Table 4 shows the prevalence of the mutation in various studies.

**Table 4:** Summary of KIT mutations described in the literature

Author	Year	Number of Patients	KIT Mutation
Beadling *et al.* [27].	2008	29	8.4%
Carvajal *et al.* [28]	2011	5	40%
Schoenewolf *et al.* [29].	2012	12	0%
Turri-Zanino *et al.* [7]	2013	32	12.5%
Zebary *et al.* [30].	2013	56	3.6%
Present study.	2015	28	25%
	Total	162	9.8%

The role of the location of the KIT mutation in HNMM has been little studied. However, its prognostic value in GIST tumors has been shown in the literature: exon 11, which codifies the juxtamembrane domain, is involved in autoinhibition of the receptor; the mutations in this juxtamembrane domain impede this inhibitory function, increasing the dimerization of the receptor, independently of its activation. This location, in turn, is the most sensitive (responds better) to treatment with tyrosine kinase inhibitors. This indicates that HNMM is potentially sensitive to tyrosine kinase inhibitors [31].

In our results, there was not one mutation more frequent than the others: 2 cases V551I, 1 case L657F, 1 case L455M, 1 case L576P, 1 case S480F, and 1 case G499S. Although the available studies comprise a small number of patients and several studies have demonstrated the wide variety of mutations (Table 5). We attempted to evaluate clinical-pathological characteristics and link them to KIT mutations. The results were discouraging, as none of the characteristics analyzed presented higher prevalence of mutation, despite the number of mutations identified being highly significant (8/28 patients, 25%).

**Table 5:** Correlation of molecular aberration

Variable	Carvajal *et al.* [28]	Guo *et al.* [8]	Hodi *et al.* [32]	Lee *et al.* [33]	Yun *et al.* [34]	Present Study
KIT mutation, n	24	40	13	27	7	7
Exon 11	9 (37.5%)	17 (42.5%)	9 (69.3%)	17 (62.9%)	5	3/7 (42.8)
Exon 13	6 (25%)	9 (22.5%)	3 (23.1%)	6 (22.2%)	1	1/7 (14.3)
Exon 9	NA	NA	NA	NA	NA	3/7 (42.8)
Exon 17	NA	NA	NA	NA	1	NA
**Specific mutation type**						
Exon 11 L576P	7 (77.8%)	NA	3 (33.3%)	5 (29.4%)	2 (40%)	1 (33.3%)
Exon 13 K642E	4 (66.6%)	NA	3 (100%)	1 (16.7%)	NA	NA
Other Mutations	No	NA	Exon 11 insetion PYD577-582 Exon 17 D820Y Exon 11 V560D	Exon 17 I817L Exon 11 deletion Exon 11 V559A	NA	Exon 11 V551 Exon 13 L657F Exon 9 L455M, S480F, G499S

NA, not available.

An interesting result was the distribution of mutations in terms of anatomical sites. In nasosinusal tumors, mutations were found in 4 cases (19% of patients), whereas in the group of patients with mouth tumors mutations were found in 3 patients (75% of patients).

Our results suggest a higher incidence of mutations in melanomas of the oral cavity. This finding is not consistent with the literature data, in which mutations are more common in tumors of the sinuses [30]. No prognostic impact caused by the presence of the KIT mutation was found in the present study. We evaluated the relationship of the mutation with OS and DFS. DFS was 28.6% in 48 months in both groups, i.e., in patients with the mutation and in those who presented the wild-type KIT (*p* = 0.771). SG was higher in patients with the wild-type gene, with a 5-year OS of 47.6%, but without statistical significance (*p* = 0.935).

KIT mutations have been evaluated in various other tumors, and in some neoplasms they are an important prognostic factor. In gastrointestinal stromal tumors (GIST), the KIT mutation is an independent risk factor for OS and DFS; this relationship is so important that exon 11 is related to a better therapeutic response. GIST tumors are an example of the importance of KIT in the selection for treatment and prognosis. In patients with leukemia, KIT mutations can suggest a greater risk of relapse [35].

However, the prognostic importance of KIT mutations in melanoma was not evaluated in a series of adequate size. Our study confirmed the presence of 25% of mutation in HNMM, thus suggesting a route to study the pathophysiology of this tumor, with a focus on the MAPKs cascade and the inclusion of patients in clinical trials with KIT inhibitors.

In conclusion, the continual evaluation of KIT mutations is essential, in an attempt to identify biomarkers and improve the selection of patients for targeted therapy.

## MATERIALS AND METHODS

For the formation of the group of this study, patients with head and neck mucosal melanomas were selected, enrolled, and treated at the Head and Neck Surgery Department of the National Cancer Institute (Instituto Nacional de Câncer - INCa). Cases without paraffin blocks of tumor tissue or cases with incomplete information on the treatment used were excluded. Molecular analyses of KIT mutations and of the pattern of response to the treatment used (surgical resection, radiotherapy, or a combination of the two) were considered for study in addition to clinical and pathology data. The study was approved by the Research Ethics Committee, under number 272/11.

### DNA extraction method

DNA was extracted from tissue and embedded and fixed in paraffin using the MagNA Pure LC 2.0 semi-automated system (Roche Applied Science), according to the manufacturer’s instructions. The procedure consisted of deparaffination with xylene, followed by graduated addition of ethane to eliminate any xylene residues.

Deparaffination with xylene was followed by digestion by proteinase K (BioAmerica, Inc, Homestead, FL, USA) using the Wizard^®^ Genomi DNA purification kit (Promega, Madison, WI, USA). The extraction was confirmed by running a 5 µL aliquot in 0.8% agarose gel in electrophoresis buffer (TBE 1X). To evaluate the integrity and concentration of the extracted DNA, a 1 µL aliquot was analyzed in a ND-1000 spectrophotometer (NanoDrop Technologies Inc., Delaware, USA).

### KIT mutation analysis

Mutational analysis of the gene was performed by Polymerase chain reaction (PCR) mplification and genome sequencing. The analysis began with exon 11 in the KIT gene, in which the majority of mutations are found (70% of the described mutations), followed by exons 9, 17, and 13. It should be emphasized that the mutations are exclusive.

Exons 9, 11, 13, and 17 of the KIT gene were amplified using the primer pairs described in Table 6. The purified PCR products were prepared for genome sequencing, using a final volume of 7.5 µl (solution containing primer, DNA, and milli-Q water). Sequencing was performed using the Sanger method, and the software used in the analysis of the results was the automated sequencer ABI PRISMTM 377 (Applied Biosystems Foster City, California, USA), which interprets the results by titrating each base in relation to the intensity of the fluorescent compound, denominating 4 specific colors. The software used in the analysis of sequencing uses the same 4 specific colors for each base. By convention, A (adenine) was labeled as green, C (cytosine) as blue, T (Thymine) as red, and G (guanine) as black, in the electropherogram image.

**Table 6:** Sequence of oligonucleotides used to amplify the KIT genes

Gene	Target	Sequence (3′-5′)
KIT	Exon 9F Exon 9R Exon 11F Exon 11R Exon 13F Exon 13R Exon 17F Exon 17R	TTCCTAGAGTAAGCCAGGGC ACAGAGCCTAAACATCCCCT CCAGAGTGCTCTAATGACTGAGAC AGGTGACATGGAAAGCCCCTG CTGCATGCGCTTTGACATCAG CTAGCATTGCCAAAATCATATT GTTTTCTTTTCTCCTCCAACCT CCTTTGCAGGACTGTCAAGC

Legend: F: Sense; R: Antisense.

### Statistical analysis

The statistical analyses were performed using the SPSS software, version 18.0 (SPSS Inc., Chicago, IL, USA). Overall survival (OS) (defined as the time between the start date of the treatment and the date of death) and disease-free survival (DFS) (defined as the time between treatment and recurrence) were estimated by the Kaplan–Meier method, and the survival curves were compared using the log-rank test. Univariate analysis was performed to evaluate the association between the variables age, sex, primary tumor site, mutational status of the KIT gene, and DFS. A *p* value ≤ 0.05 was considered statistically significant.
